# Non-Targeted Metabolomics Analysis of Metabolic Differences Between Different Concentrations of Protein Diets in the Longest Dorsal Muscle of Tibetan Pigs

**DOI:** 10.3390/metabo15080555

**Published:** 2025-08-19

**Authors:** Feifan Zhang, Jinhui Liang, Hongliang Zhang, Mengqi Duan, Dong Yang, Chamba Yangzom, Peng Shang

**Affiliations:** 1 College of Animal Science, Xizang Agriculture and Animal Husbandry University, Linzhi 860000, China; 17654540263@163.com (F.Z.); ling565890@126.com (J.L.); zhanghongliang@xza.edu.cn (H.Z.); zduanduan0117@163.com (M.D.); m15086795164@163.com (D.Y.); 2 Provincial-Ministerial Collaborative Innovation Center for R&D of Agricultural and Animal Husbandry Resources with Tibetan Characteristics, Linzhi 860000, China; 3 Key Laboratory of Tibetan Pig Genetic Improvement and Reproduction Engineering, Linzhi 860000, China

**Keywords:** Tibetan pig, longest back muscle, dietary protein level, LC-MS analysis, off-target metabolism

## Abstract

**Background/Objectives:** The aim of this study was to explore the effects of diets with different protein levels on the metabolite composition and metabolic pathways of the longest dorsal muscle of Tibetan pigs, in order to provide a metabolic basis for optimizing the nutritional regulation strategy of Tibetan pigs. **Methods:** A total of 32 healthy 180-day-old depopulated male Tibetan pigs were randomly divided into four groups and fed diets with protein levels of 10%, 12%, 14%, and 16%, respectively, with a feeding cycle of 8 weeks. The longest dorsal muscle samples were collected, and their metabolic profiles were systematically analyzed by LC-MS non-targeted metabolomics. **Results:** The TIC plots of the quality control samples were highly overlapped, indicating a stable instrumental detection process and good consistency of sample processing. Principal component analysis and orthogonal partial least squares discriminant analysis revealed significant metabolic differences between groups with different protein levels. A total of multiple differential metabolites was obtained based on VIP value and *p*-value screening, and Venn diagram analysis revealed a total of 11 metabolites among the three comparative groups, suggesting that they may have key roles in the protein regulation process. Volcano plots further clarified the number and trend of significantly up- and down-regulated metabolites in each group. KEGG pathway enrichment analysis showed that, with the elevation of protein level, the metabolic pathway response showed a tendency of expanding from basal energy metabolism to the complex network of amino acid synthesis, steroidogenesis, endocrine signaling, and detoxification pathways, especially in the high-protein-treated group. **Conclusions:** The study showed that different protein intake levels could significantly regulate the metabolites and key metabolic pathways in the longest muscle of Tibetan pigs, which provided theoretical support for the scientific formulation of a protein supply program to optimize the quality and growth performance of Tibetan pork.

## 1. Introduction

Pig is one of the most important livestock and poultry economic animals globally, belonging to the order of even-toed ungulates, which is widely farmed for its excellent meat quality, short growth cycle, and strong reproductive performance and has traditionally been an important source of meat, leather, and other by-products for human beings [1]. In the Tibetan Plateau region of western China, the Tibetan pig, as a typical local pig breed, has formed unique genetic characteristics and ecological adaptability after long-term natural domestication and environmental adaptation [2,3,4]. The Tibetan pig is mainly distributed in the plateau zone with an altitude of more than 4000 m, and its living environment is generally characterized by low oxygen, low temperature, strong ultraviolet radiation, and poor forage resources [5]. During the long evolutionary process, the Tibetan pig has gradually developed a high degree of adaptability to the extreme environment of the plateau at the morphological, physiological, and metabolic levels, showing advantages such as high resistance to cold and disease, stable reproductive performance, and suitability for grazing [6,7,8]. At the same time, Tibetan pigs have long provided meat, skin, hair, and other production and living materials for Tibetan farmers and herdsmen, occupying a central position in the agricultural and animal husbandry system on the Tibetan Plateau. However, compared with modern commercial pig breeds (e.g., Long White, Large White, Duroc, etc.), the Tibetan pig has disadvantages, such as slow growth rate, low feed conversion rate, and low carcass leanness, and its average daily weight gain is significantly low [9,10], which seriously restricts the improvement of the economic benefits of the Tibetan pig breeding industry and the development process of its industrialization. Therefore, under the premise of protecting its excellent local traits, enhancing the growth performance and meat production efficiency of Tibetan pigs through scientific nutritional interventions has become a key research direction to promote its sustainable development and industrialization.

As an essential nutrient for animal growth and development, tissue repair, and metabolic maintenance, protein has a central regulatory role in growth rate, organ development, immune level, and muscle synthesis in livestock and poultry animals [11,12,13]. For pigs, the longest dorsal muscle is the largest and most economically valuable muscle tissue in the carcass, and its growth rate and degree of muscle fiber development directly determine the yield and quality of pork [14,15]. Recent studies have shown that the protein concentration in the ration has a significant effect on the growth and metabolism of the longest dorsal muscle in pigs [16]. Appropriate protein levels can not only increase the number and diameter of muscle fibers by regulating myo-satellite cell proliferation and differentiation [17], but also promote muscle protein synthesis and muscle tissue remodeling by modulating classical signaling pathways such as PI3K-Akt/mTOR [18,19]. However, studies on the effects of protein feeds on muscle growth and metabolism in Tibetan pigs are extremely limited, and most of the relevant studies focus on common commercialized pig breeds, such as Duroc or Large White pigs, but lack in-depth and systematic analysis of the physiological and metabolic characteristics specific to the longest dorsal muscle of Tibetan pigs. Most of the studies were based on growth performance or blood biochemical indexes, but lacked systematic research on the changes in endogenous metabolites and the metabolic network involved in muscle tissue, especially the dynamic changes in metabolites and their regulatory mechanisms in the longest muscle of Tibetan pigs under different protein feeding conditions. This research gap not only limits the precise optimization of protein nutritional intervention strategies, but also hinders the scientific construction of an efficient feeding model for Tibetan pigs.

Metabolites are the end products of various biochemical reactions in organisms and are the important material basis for the formation of phenotypic traits [20]. Metabolomics, as one of the rapidly developing biology technology systems in recent years, is dedicated to the comprehensive, high-throughput, qualitative, and quantitative analysis of metabolites produced by organisms under specific physiological states or environmental disturbances, in order to reveal their physiological regulatory mechanisms, stress response mechanisms, and the basis of disease occurrence [21]. Metabolomics research is mainly categorized into targeted and non-targeted modes, among which non-targeted metabolomics technology has the advantages of wide coverage, high data dimension, and a strong exploratory nature and has been widely used in the research fields of nutritional regulation, disease diagnosis, animal genetic breeding, and environmental response [22,23,24]. Non-targeted metabolomic studies are usually performed by liquid chromatography–mass spectrometry (LC-MS) to compare and analyze metabolic profiles between experimental and control groups, from which statistically significant differential metabolites are screened out, and the metabolic pathways are further enriched by databases to explore the roles of these metabolites in specific biological processes [25,26]. Therefore, in this study, we proposed to systematically analyze the metabolite composition of the longest dorsal muscle of Tibetan pigs under different protein concentration feeding conditions using non-targeted metabolomics technology based on a high-resolution mass spectrometry platform. By constructing screening models of differential metabolites and metabolic pathway enrichment analysis, we systematically reveal the potential regulatory mechanisms of protein level changes in muscle metabolic processes in Tibetan pigs.

## 2. Materials and Methods

### 2.1. Laboratory Animals and Husbandry Management

In this study, 32 healthy 180-day-old deprived male Tibetan pigs from local Tibetan sources were selected and randomly divided into four groups of 8 pigs each, named the ALD, BLD, CLD, and DLD groups, respectively. The four groups were fed isoenergetic rations with crude protein levels of 10%, 12%, 14%, and 16%, respectively, to study the effects of different protein concentrations on the metabolic characteristics of the longest dorsal muscle of Tibetan pigs. The diets consisted of corn, wheat bran, soybean meal, calcium carbonate, calcium phosphate, lysine hydrochloride (Lys-HCl), DL-methionine (DL-Met), DL-threonine (DL-Thr), tryptophan (Trp), salt, choline, multivitamins, mineral additives, and diatomaceous earth, and the formulas fulfilled the nutrient requirements of nursery pigs in the Standard for Swine Feeding (NY/T65-2004) requirements in the Standard of Pig Feeding (NY/T65 2004) (Table 1). The experimental period was 8 weeks. During the experimental period, all pigs were individually housed in standard metabolic observation cages (1.2 m × 0.8 m) under a constant environment with temperature control of 22 ± 1 °C and relative humidity of 60 ± 5%. The pigs were allowed to feed and drink freely, and the whole experimental process was carried out in strict accordance with the norms of animal welfare.

### 2.2. Longest Dorsal Muscle Sample Collection

At the end of the 8-week feeding experiment, all Tibetan pigs were fasted for 12 h before slaughtering, and the slaughtering process strictly followed the relevant provisions of the Regulations on the Management of Laboratory Animals. After slaughter, the longest dorsal muscle (located between the 10th and 13th ribs) was quickly removed within 30 min, and then about 1.5 g of tissue sample was extracted with sterile scissors and forceps. The collected muscle tissue of the longest dorsal muscle was quickly placed in a pre-cooled 2 mL centrifuge tube, quick-frozen in liquid nitrogen for 5 min, and then transferred to a refrigerator at −80 °C for storage, to be used for subsequent metabolomics analysis. Each sample was numbered, and its corresponding individual information and feed group were indicated to ensure accurate data.

### 2.3. Reagents and Instruments

Methanol, acetonitrile (all chromatographic grades, Merck, Darmstadt, Germany); acetic acid (all chromatographic grades, Ron, Shanghai, China), ammonium formate, ammonia, carboxylic acid (all chromatographic grades, Aladdin, Shanghai, China). Mass spectrometer (Q Exactive HF-X, Thermo Scientific, Waltham, MA, USA); ultra-high-performance liquid (Vanquish, Thermo Scientific, Waltham, MA, USA); chromatograph (5424R, Eppendorf, Hamburg, Germany); centrifuge concentrator (CentriVap, LABCONCO, Missouri Kansas, MO, USA); automated workstations (Biomek i5, Beckman Coulter, Brea, CA, USA).

### 2.4. Metabolite Extraction

Frozen muscle samples were removed from the −80 °C refrigerator and thawed on ice, and all subsequent operations were conducted on ice. After the samples were thoroughly ground in liquid nitrogen, about 20 mg (±1 mg) of powder was weighed into pre-numbered centrifuge tubes, and 400 μL of 70% methanol/water mixture extract containing the internal standard was added, and the samples were shaken at 251× *g* for 5 min and then left on ice for 15 min. Subsequently, centrifugation was performed at 16,000× *g* for 10 min at 4 °C, and 300 μL of the supernatant was transferred to a new numbered centrifuge tube and allowed to stand at −20 °C for 30 min. After centrifugation again at 16,000× *g* for 3 min at 4 °C, 200 μL of the supernatant was transferred to the liner tube of the injection bottle for subsequent metabolomics analysis.

### 2.5. LC-MS Analysis

#### 2.5.1. Chromatograph

Column: Waters ACQUITY Premier HSS T3 Column 1.8 µm, 2.1 mm × 100 mm; mobile phase A: 0.1% (*v*/*v*) formic acid/water; mobile phase B: 0.1% (*v*/*v*) formic acid/acetonitrile; column temperature: 40 °C; flow rate: 0.4 mL/min; Injecting volume: 4 μL; elution: gradient elution. The gradient elution program has a total duration of 10 min and uses two mobile phases: a 0.1% (*v*/*v*) formic acid aqueous solution for phase A and an acetonitrile solution containing 0.1% (*v*/*v*) formic acid for phase B. The gradient elution program is performed at the start of the program (0.0 min). At the start of the program (0.0 min), the proportion of phase A was 95%, and the proportion of phase B was 5%. Within 2.0 min, the phase A ratio linearly decreased to 80% while the phase B ratio increased to 20%. Subsequently, over the next 3.0 min (to 5.0 min), the phase A ratio continued to decrease linearly to 40% while the phase B ratio rose to 60%. Then within 1.0 min (to 6.0 min), the A phase proportion rapidly decreased to 1%, and the B phase proportion rose to 99% and remained at this proportion for 1.5 min (to 7.5 min). Finally, within 0.1 min (to 7.6 min), the mobile phase proportions rapidly returned to their initial state (95% for phase A and 5% for phase B) and were maintained at this ratio until the end of the program (10.0 min).

#### 2.5.2. Mass Spectrometry Conditions

Operating parameters of the Q Exactive HF-X mass spectrometer in positive ion mode (ESI+) and negative ion mode (ESI−). The two modes share the same settings: sheath gas flow rate of 30 Arb, auxiliary gas flow rate of 5 Arb, ion transfer tube temperature of 320 °C, and nebulization temperature of 300 °C; in MS1 (primary mass spectrometry) both have mass scanning ranges of 75–1000 Da, resolutions of 35,000, and automatic gain control (AGC) target values of 1 × 10^6^; the collision energy step (Collision Energy Step) is 50 V; in MS2 (secondary mass spectrometry) both have collision energy steps of 50 V; and in MS2 (secondary mass spectrometry) both have collision energy steps of 50 V. The two modes have the same operating parameters. The key difference between the two modes is the ionization voltage (spray voltage), which is 3500 V for the positive ion mode and 3200 V for the negative ion mode.

### 2.6. Data Processing and Statistical Analysis

The raw data from the mass spectrometer were converted to MZML format by ProteoWizard, and the XCMS program was used for peak extraction, alignment, and retention time correction. The specific practice was to match the mz error between the primary (Q1 from full-scan scanning) and the secondary (Q1 from product ion scanning) to be less than 25 ppm, and to match the rt error between the primary (Q1 from full-scan scanning) and the secondary (Q1 from product ion scanning) to be 6 s. The corrected and screened peaks were searched through the laboratory’s self-constructed databases, The corrected and screened peaks were identified by searching the laboratory’s own database, integrating public libraries, prediction libraries, and the metDNA method for metabolite identification. Data matrices were imported into SIMCA (v14.1, Umetrics, Umeå, Sweden) software for multivariate statistical analysis, including principal component analysis and orthogonal partial least squares discriminant analysis. Variable importance in the projection (VIP) values for each metabolite were calculated using the OPLS-DA model and combined with one-way analysis of variance (ANOVA) to screen out significantly different metabolites with VIP > 1 and *p* < 0.05. The biological significance of the differential metabolites was analyzed by metabolic pathway enrichment through the KEGG (Kyoto Encyclopedia of Genes and Genomes) database to screen the significant pathways related to protein nutritional regulation. Pathway enrichment was performed using a hypergeometric test combining the pathway enrichment factor (Rich Factor) and the *p*-value to visualize the results.

## 3. Results

### 3.1. LC-MS Data QC and Total Spectrum Characterization

**Figure 1:**
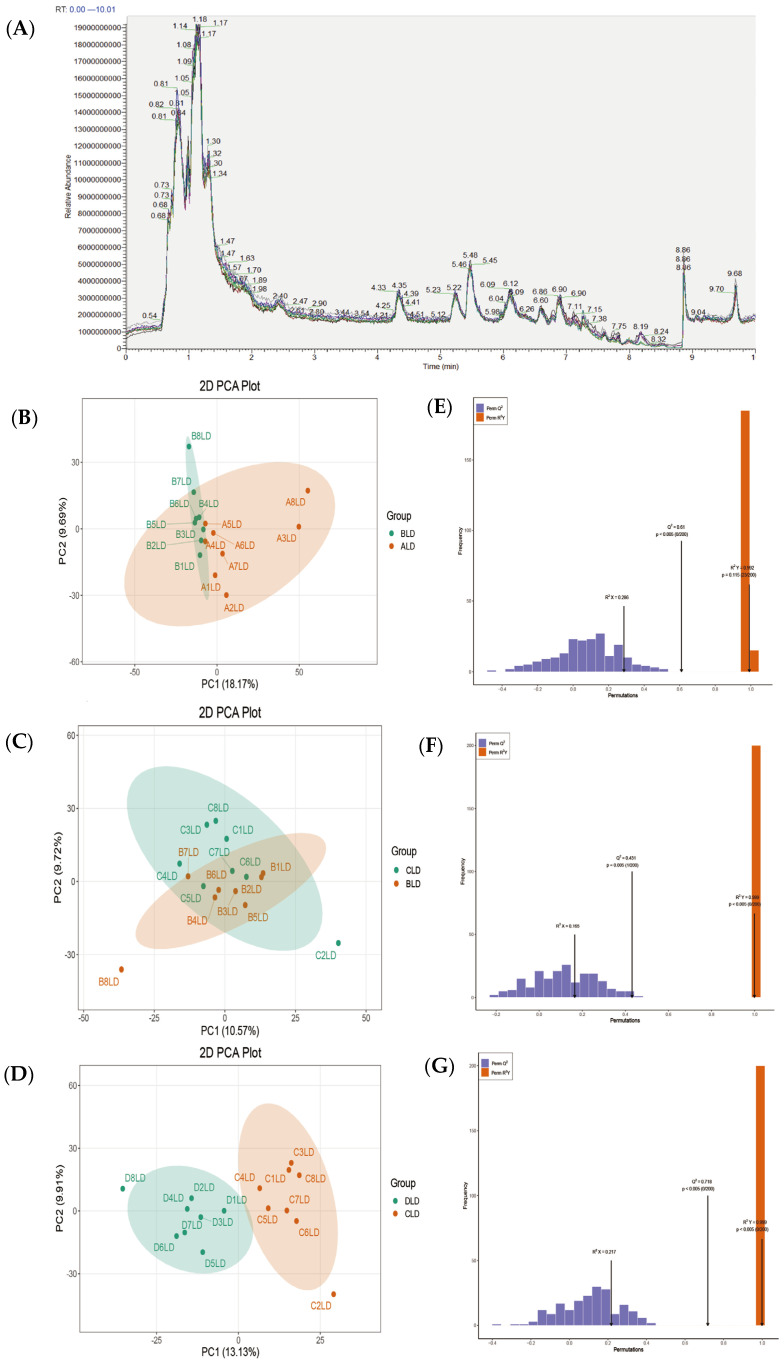
Overlay of TIC for mass spectrometry detection of QC samples (**A**). Differences in metabolite distribution across protein groups, plots of PCA scores of mass spectrometry data of LD samples and quality control samples (**B**–**D**), OPLS-DA validation plots of the longest dorsal muscles of Tibetan pigs in each group (**E**–**G**).

In order to evaluate the technical reproducibility of the samples under the same process, a quality control (QC) sample was set up in this study, which was prepared by mixing the extracts of each experimental group in equal volume to monitor the fluctuation of the instrument and the reproducibility of the data. During the LC-MS assay, one QC sample was inserted for every 10 samples analyzed to monitor the stability and reproducibility of the assay process in real time. The overlap analysis of TICs of different QC samples showed good overlap in retention time and peak intensity without abnormal drift or loss of signal, which indicated that the instrument was in stable condition, the quality of data acquisition was good, and the metabolites were extracted and detected in good technical consistency (Figure 1A).

**Figure 2:**
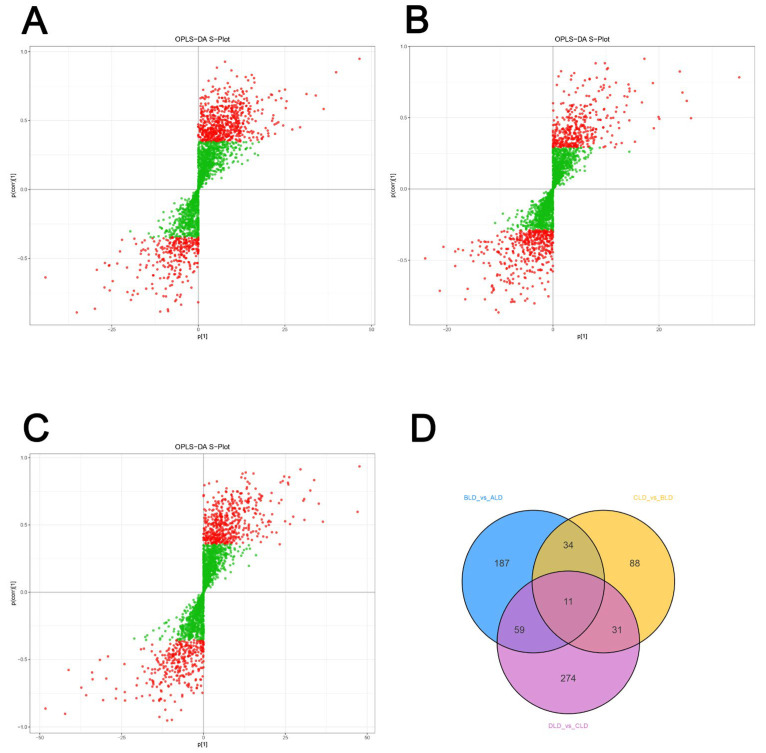
OPLS-DA S-plot plots (**A**–**C**),Wayne diagram of LD differences (**D**).

In order to investigate the effects of diets with different protein levels on the metabolite composition of the longest dorsal muscle of Tibetan pigs, the metabolic profile data of each group of samples were analyzed by principal component analysis (PCA) and orthogonal partial least squares discriminant analysis (OPLSDA) to reduce the dimensionality of the metabolic profiles and differentiate the data between groups. The PCA results showed that samples of each group were more dispersed in the principal component space, which indicated that there was a certain difference in overall metabolic profiles among the treatments with different protein levels. Meanwhile, the QC samples were tightly clustered, which verified the stability of the detection system and the reliability of the data (Figure 1B–D). Further modeling analysis of intergroup differences by OPLS-DA showed that there was a clear distinction in metabolite composition among the four groups of samples (Figure 1E–G). The model R^2^X, R^2^Y, and Q^2^ values were all greater than 0.5, indicating good model fit and strong predictive ability. Through the model loading diagram and VIP (variable importance in projection) analysis, the metabolite characteristic peaks with significant contributions among different protein treatments were preliminarily screened out (Figure 2A–C). These results indicated that changes in dietary protein levels significantly affected the metabolic profiles of the longest dorsal muscle of Tibetan pigs, laying the foundation for subsequent differential metabolite screening and pathway function annotation.

### 3.2. Differential Metabolite Identification

**Figure 3:**
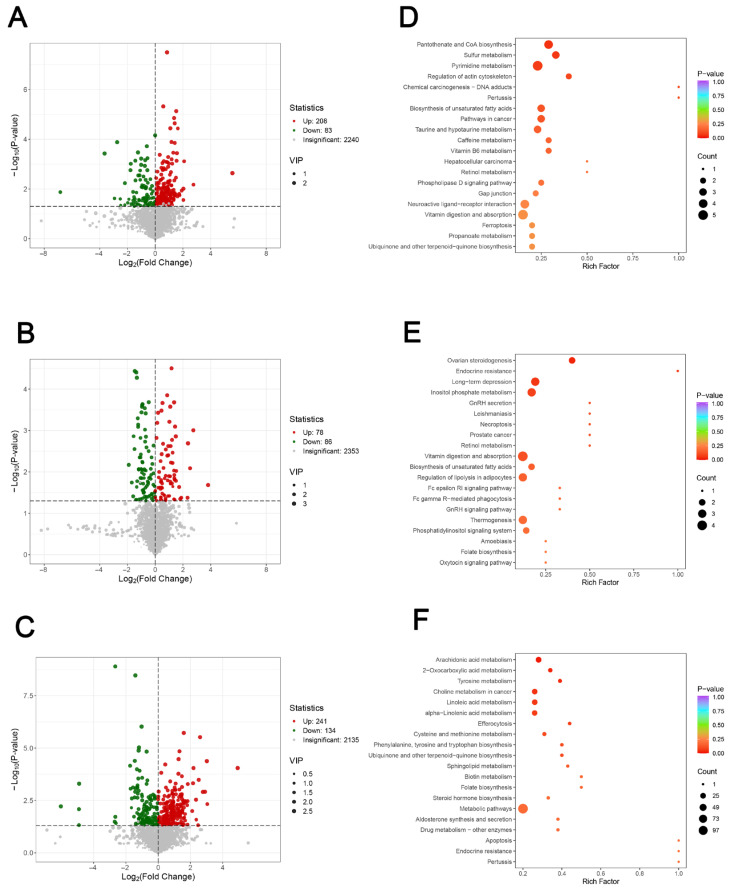
Volcano plot of LD differential metabolites (**A**–**C**), KEGG differential metabolite pathway enrichment map (**D**–**F**).

In order to screen the differential metabolites in the longest dorsal muscle of Tibetan pigs under different protein level treatments, this study was based on the VIP values calculated by the OPLS-DA model and the results of the one-way analysis of variance (ANOVA), and the metabolites that were statistically significant and had a high degree of interpretation in the model were screened out using the criteria of VIP > 1 and *p* < 0.05 as the determination criteria. The intersection analysis of differential metabolites among the four groups was performed using Venn diagrams (Figure 2D), and a total of the multiple differential metabolites was screened out by two-by-two comparisons of the treatment groups with different protein levels (BLD_vs._ALD, CLD_vs._BLD, DLD_vs._CLD). Wayne diagram analysis revealed significant metabolic-specific variations between groups, with a total of 11 differential metabolites appearing simultaneously in all three group comparisons, suggesting that they may have a key role in the regulation of muscle metabolism induced by changes in protein intake. These metabolites, covering ketones, aldehydes, phospholipids, aromatic acids, small-molecule peptides, and glucose metabolites, have a wide range of biological functions and may be involved in important processes such as cell signaling, membrane lipid metabolism, and redox homeostasis (Table 2). The volcano diagram was further used to visualize the distribution of up-regulated and down-regulated significant metabolites among the groups (Figure 3A–C). In the volcano diagram, the significantly different metabolites (*p* < 0.05, |log2 Fold Change| > 1) showed a clear distribution trend, and the metabolites that were significantly different between the treatment groups with different protein concentrations were screened out. The results showed that ALD vs. BLD screened a total of 291 metabolites, of which 208 were up-regulated and 83 were down-regulated; BLD vs. CLD screened a total of 164 metabolites, of which 78 were up-regulated and 86 were down-regulated, and CLD vs. DLD screened a total of 375 metabolites, of which 241 were up-regulated and 134 were down-regulated, which shows that the DLD group has the highest number of differential metabolites and has a larger number of differential metabolites than the other groups with the largest differences. The above results indicated that the response range of metabolites in the longest dorsal muscle of Tibetan pigs expanded with the gradual increase in dietary protein content, and the metabolic changes were more dramatic, especially in the stage of higher protein level (CLD vs. DLD), suggesting that the high protein intake may cause a greater degree of metabolic remodeling.

### 3.3. Analysis of Key Metabolic Pathways

To further clarify the effects of different protein intake levels on the metabolic network of the longest dorsal muscle of Tibetan pigs, the present study was conducted to analyze the KEGG pathway enrichment of the differential metabolites screened in each group and to draw bubble plots to assess the functional localization and the degree of enrichment in the top 20 metabolic pathways. The results showed that the pathway distributions of metabolites in different comparison groups both showed some overlap and exhibited specific response patterns. The differential metabolites in the ALD_vs._BLD group were analyzed for KEGG pathway enrichment. The analysis results showed (Figure 3D) that these metabolites were mainly involved in several physiological regulatory modules, such as energy anabolism, amino acid transformation, lipid metabolism, antioxidant mechanisms, vitamin transformation, and signaling pathways, revealing a wide range of metabolic adaptive processes in muscle tissues against the background of high nutrient input. In terms of energy metabolism, the pantothenic acid and coenzyme A synthesis pathways were the most significantly enriched. Coenzyme A is a key metabolic factor linking carbohydrate, fatty acid, and amino acid metabolism [27], and its enhanced synthesis may have provided the tissues with higher energy conversion capacity to support the metabolic load associated with increased protein intake [28]. Meanwhile, the activation of the sulfur metabolic pathway suggests elevated utilization of glutathione synthesis-related substrates, such as cysteine, suggesting that the antioxidant system is in a stress-responsive state [29,30]. Enrichment of the pyrimidine metabolic pathway suggests increased cellular nucleotide requirements, which may involve active RNA synthesis, cell proliferation, or repair mechanisms [31]. In lipid metabolism, enrichment of unsaturated fatty acid biosynthesis pathways reveals a regulatory need for muscle cells in terms of membrane structure renewal, signaling lipid synthesis and inflammatory homeostasis. In addition, the enrichment of retinol metabolism and vitamin uptake pathways further revealed that the activation and transport of fat-soluble vitamins such as vitamin A are critical in metabolic remodeling, and retinoic acid, in particular, plays a significant role in the regulation of the muscle stem cell state, immune regulation, and antioxidants [32]. Of particular interest, the iron death pathway, a form of programmed cell death characterized by iron ion dependence and lipid peroxidation, which is usually activated under stressful conditions, also showed a trend of significant enrichment [33]. The manifestation of this pathway in the present study may indicate that in high-protein environments, the level of cellular lipid oxidation rises and needs to rely on antioxidant mechanisms to maintain homeostasis. In addition, disease-related metabolic pathways, such as hepatocellular carcinoma, cancer-associated pathways, and DNA adduct formation, were also shown in the enrichment maps. Although the appearance of such pathways may be related to the overlap of the KEGG database annotations, it may still indicate that some metabolites are involved in DNA modification, cell cycle, or stress-related mechanisms, which is worth further analyzing in conjunction with the information on specific metabolites.

The results in the BLD_vs._CLD group indicated that the metabolites were mainly enriched in multiple signaling pathways with well-defined biological functions, including neuro-endocrine regulation, lipid and vitamin metabolism, regulation of energy homeostasis, cellular signaling, and immune response-related pathways (Figure 3E). First, among the neuro-endocrine regulatory pathways, the significant enrichment of the gonadotropin-releasing hormone secretion pathway and the GnRH signaling pathway indicated that further elevation of protein levels may affect reproductive function and sex hormone synthesis by intervening in the regulation of the hypothalamic–pituitary–gonadal axis. The activation of the signaling pathway of GnRH, which is upstream of the regulation of the secretion of luteinizing and follicle-stimulating hormone [34], indicated that the muscle tissue may be regulated by endocrine feedback in an indirect manner under moderate protein conditions. In addition, the enrichment of the oxytocin signaling pathway indicated that this pathway may be involved in processes such as energy uptake behavior, smooth muscle regulation, or anti-stress response in mammals, indirectly reflecting the coupling between protein intake and neurohormonal activities [35], and this enrichment revealed a significant adaptive response of muscle tissues in terms of endocrine, nutrient uptake, and metabolic coordination after elevation of the dietary protein level from low to medium. Sustained metabolite enrichment was also observed in the ovarian steroidogenic pathway, which further supports the above endocrine regulatory trend. Lipid metabolic pathways also showed high enrichment, mainly including unsaturated fatty acid biosynthesis and lipolytic regulation in adipocytes. The former involves cell membrane construction, signaling molecule generation, and anti-inflammatory mechanisms, while the latter is closely related to energy mobilization and lipid storage regulation [36]. The results showed that moderate protein intake was sufficient to activate multilevel lipid metabolic remodeling mechanisms in the longest muscle of the back, suggesting that the body adapts to the new nutritional environment while enhancing metabolic efficiency through lipid plasticity.

The results in the CLD_vs._DLD group showed (Figure 3F) that these metabolites were widely enriched in the key metabolic pathways of fatty acid metabolism, amino acid biosynthesis, steroid hormone synthesis, mitochondrial energy metabolism, and signaling, and several of them were enriched to a significant degree, indicating that they were of great biological significance under the current nutritional state. At the level of lipid metabolism, arachidonic acid metabolism, linoleic acid metabolism, and α-linolenic acid metabolism showed significant enrichment, and these pathways play a central role in regulating the composition of cell membrane phospholipids, the synthesis of inflammatory mediators, and signal transduction lipid production, and their metabolites may reflect that muscle tissues can promote the remodeling of membrane lipids, improve the efficiency of cell mobility and signaling, and further enhance the adaptive capacity of tissues to environmental changes under the condition of the adequate protein supply organization’s ability to adapt to environmental changes. For amino acid metabolism, multiple pathways were observed to be significantly enriched in tyrosine metabolism, cysteine and methionine metabolism, and phenylalanine, tyrosine, and tryptophan biosynthesis. These metabolic pathways are not only involved in the direct construction of protein structures, but also mediate signal transduction (e.g., dopamine, epinephrine, sulfur-based antioxidants) through their derived metabolites, suggesting that after up-regulation of protein uptake levels, muscle tissues may have enhanced their ability to transform, store, and regulate amino acids to adapt to continuous anabolic states and metabolic stress. In steroid hormone-related metabolism, the enrichment of steroid hormone biosynthesis with the aldosterone synthesis and secretion pathway reflects the possibility that endocrine signaling may be activated by the high-protein state. Steroid hormones have important roles in the regulation of electrolyte homeostasis, protein synthesis, and anti-inflammatory responses [37,38], and the activation of their metabolic pathways may signal the regulation of water–salt homeostasis and protein anabolic processes in muscle tissues through endocrine mechanisms. In addition, the enrichment of metabolic pathways related to ubiquinone and other terpene quinones synthesis, the mitochondrial electron transport chain, and the enrichment of metabolic pathways may indicate that mitochondrial metabolic activity is elevated under high-protein conditions, which is conducive to the enhancement of ATP synthesis, the improvement of cellular energy supply, and the maintenance of metabolic homeostasis [39]. Immunity and programmed death-related pathways such as phagocytosis of apoptotic cells, the apoptotic pathway, and drug metabolism were also detected as a trend of enrichment. Muscle tissues may be accompanied by mild inflammatory responses, removal of metabolic wastes, and cellular renewal in the context of high metabolism, to maintain stable tissue function. Notably, although the annotated pathways such as choline metabolism, the cancer-related signaling pathway, and endocrine resistance do not necessarily reflect the disease state directly, their metabolites are involved in key processes such as phospholipid synthesis, nucleic acid modification, and hormone signaling regulation, and may be indirectly involved in the regulation of muscle tissues in response to enhanced protein supply.

## 4. Discussion

In this study, we systematically analyzed the effects of different dietary protein levels (10%, 12%, 14%, and 16%) on the metabolic profiles of the longest dorsal muscle of Tibetan pigs by using non-targeted metabolomics and revealed the systematic effects of protein nutrition on the regulation of skeletal muscle metabolism in several dimensions, such as screening for differential metabolites, multivariate statistical analysis, and pathway enrichment. The results showed that the types of metabolites and their abundance in the longest dorsal muscle changed significantly with the increase in dietary protein level, presenting a strong protein response profile.

First, from the results of principal component analysis and OPLS-DA, there was a clear distinction between the samples in each group in terms of metabolic composition, and the QC samples were tightly clustered, indicating that the experimental detection system was stable and reliable. The results of differential metabolite screening showed that the number of differential metabolites gradually increased from low to high protein levels (ALD → DLD), especially up to the 375 metabolites that were screened in the CLD vs. DLD group, indicating that the driving effect of high protein intake on metabolic remodeling of skeletal muscle was the most significant. This trend indicated that the metabolic regulation of the longest dorsal muscle of Tibetan pigs, as a more nutrient-sensitive tissue, may activate more complex regulatory pathways with increasing nutrient density. Wayne diagram analysis showed that despite the wide variation in metabolites between groups, a total of 11 metabolites were present in all comparisons, covering a wide range of structural types, such as ketones, aldehydes, phospholipids, small peptides, and glycans. These metabolites, such as phosphocholine and erythrose, have important physiological roles in energy metabolism, membrane lipid synthesis, and cell signaling regulation and may be key molecules in the regulation of muscle metabolism triggered by changes in protein intake [40]. Huang et al. [41] showed that enhanced phosphocholine metabolism is essential for terminal erythropoiesis and is involved in the regulation of cell membrane phospholipid synthesis and metabolism. In the present study, phosphocholine may similarly affect the structure and function of muscle cells through the regulation of membrane lipid metabolism and thus participate in the regulation of muscle metabolism triggered by changes in protein intake. Volcano plots further visualized the metabolic expression trends among different protein groups, clearly indicating that the most significantly up-regulated metabolites were found in the CLD vs. DLD group, in line with the aforementioned conclusion that high protein induces greater metabolic remodeling. The KEGG pathway enrichment results provided insight into the functional modules involved in the differential metabolites and their physiological regulatory networks. At the level of energy metabolism, the enriched pantothenic acid and coenzyme A biosynthesis pathways suggest that muscle tissue meets the increased demand for ATP and intermediary metabolites in the state of high protein intake by enhancing the ability of energy substrates (e.g., fatty acids versus amino acids) to enter the tricarboxylic acid cycle. Research by Katarzyna et al. [42] on the consequences of impaired coenzyme A metabolism revealed that pantothenate kinase deficiency leads to abnormal F-actin organization, associated with abnormally elevated levels of phosphorylated cofilin within cells. This deficiency also impairs the ability of human neuronal SHSY-5Y cells to form neurites. These findings highlight the importance of coenzyme A metabolism in cells and may have implications for understanding energy metabolism problems that could arise in muscle cells when abnormalities occur in the pantothenate and coenzyme A biosynthesis pathways. Also, the enrichment of mitochondria-related metabolic pathways such as ubiquinone synthesis and thermogenesis suggests that the cellular oxidative phosphorylation capacity is activated, strengthening the energy supply system. As for lipid metabolism, it was found that the pathways of unsaturated fatty acids, linoleic acid, α-linolenic acid, and arachidonic acid metabolism were widely enriched and that muscle cell membrane lipid composition, signaling lipid synthesis, and lipolytic mobilization processes were regulated by proteotropic regulation. These changes in lipid metabolism may not only affect cellular structural stability, but also participate in the regulation of inflammation, immunity, and redox homeostasis through lipid mediators [43]. In addition, adipocyte lipolysis regulatory pathways were significantly activated, reflecting the extensive mobilization of lipids as a secondary energy source under elevated protein levels. Regarding amino acid metabolism, the present study observed a significant enrichment of aromatic amino acid metabolic pathways such as phenylalanine and tyrosine with elevated protein levels, and foreign studies have shown that excess amino acids may promote protein synthesis through mTORC1 signaling but also increase mitochondrial reactive oxygen species generation [44]. The activation of the iron death pathway in this study may be related to this, while the enrichment of the sulfur metabolic pathway suggests that the organism may enhance antioxidant capacity through glutathione synthesis, which is consistent with the antioxidant mechanism of sulfur amino acids observed in an aged mouse model [45]. In terms of vitamin and micronutrient regulation, vitamin B6 metabolism, retinol metabolism, and the vitamin uptake pathways showed significant enrichment, indicating enhanced utilization and dependence of protein intake on coenzymatic vitamins. In particular, the metabolic pathway of retinol was significantly enriched in several comparison groups, suggesting that it may play an important role in maintaining muscle stem cell differentiation, modulating immune responses, and combating oxidative stress. In addition, the enrichment of several neuroendocrine-related pathways, such as the GnRH signaling pathway, oxytocin signaling pathway, and steroid hormone synthesis and aldosterone secretion pathway was also observed, suggesting that the protein intake may indirectly affect the regulation of muscle anabolic and metabolic rhythms through the activation of the hypothalamic–pituitary–gonadal axis and steroid metabolism. This finding further demonstrates that muscle tissue not only serves as a “terminal” for energy and synthesis, but is also closely related to systemic endocrine regulation.

Overall, the KEGG pathway enrichment results fully demonstrated that changes in protein levels have profound effects on the metabolic network of the longest dorsal muscle, which involves systematic responses of multiple metabolic axes. These changes are not only reflected in the enhancement of energy synthesis and nutrient transport, but are also accompanied by the simultaneous adjustment of endocrine regulation, immune adaptation, and cellular homeostasis maintenance mechanisms, suggesting that high protein intake may play a key role in optimizing muscle metabolism and enhancing physiological functions in animals. In the future, we can further integrate transcriptomics and proteomics to analyze the core regulatory nodes and functional molecules in these key pathways, so as to reveal the deep mechanism of the protein nutritional regulation of muscle metabolism.

## 5. Conclusions

In summary, this study comprehensively revealed the systematic effects of different protein concentration levels on the metabolome composition of the longest dorsal muscle of Tibetan pigs by means of untargeted metabolomics. It was found that with the gradual increase in protein intake level, the range of metabolic responses in muscle tissue was significantly expanded, and the metabolic pathway migrated from basic nutrient metabolism to complex signal regulation and structural remodeling. In particular, the fatty acid metabolism, amino acid biosynthesis, steroid hormone synthesis, and mitochondrial energy metabolism pathways were significantly activated in the high-protein-treated group, suggesting that high-protein feeding can deeply remodel the metabolic network of Tibetan pig skeletal muscle. In this study, the regulatory effect of protein intake on muscle metabolism was systematically verified for the first time in Tibetan pigs, a local pig breed with high-plateau characteristics, which provides a solid metabolic foundation and theoretical support for nutritional level regulation and high-quality production of Tibetan pigs and also provides important clues for the subsequent development of targeted regulation, functional validation, and nutritional precision intervention.

## Figures and Tables

**Table 1:** Ration composition and nutrient levels (g).

Raw Materials	10% (ALD)	12% (BLD)	14% (CLD)	16% (DLD)
corn	257.4	232.93	208.46	184
wheat bran	120.6	125.33	130.07	134.8
soybean (oil) meal	5.2	26.67	48.13	69.6
talc	3.48	3.56	3.64	3.72
calcium biphosphate	1.12	0.75	0.37	0
Lys-HCl	3.68	2.92	2.16	1.4
DL-Met	0.84	0.63	0.41	0.2
DL-Thr	1.6	1.25	0.91	0.56
Trp	0.36	0.24	0.12	0
edible salt	1.2	1.20	1.20	1.2
choline	0.4	0.40	0.40	0.4
multidimensional	0.12	0.12	0.12	0.12
limestone	0.8	0.80	0.80	0.8
silica earth	3.2	3.20	3.20	3.2
add up the total	400	400	400	400

**Table 2:** Differential metabolites between the three LD groups.

Metabolites	Class I	Formula
Dihydro-3-methyl-2(3H)-furanone	Aldehyde, Ketones, Esters	C_5_H_8_O_2_
Phosphocholine	Organic acid and its derivatives	C_5_H_15_NO_4_P+
4,4’-Sulfonyldiphenol	Benzene and substituted derivatives	C_12_H_10_O_4_S
4-(Aminomethyl)benzoic acid	Organic acid and its derivatives	C_8_H_9_NO_2_
Benzyln-[(2 s)-4-methyl-1-[[(2 r)-4-methyl-1-[[(2 s)-4-methyl-1-oxopentan-2-yl]amino]-1-oxopentan-2-yl]amino]-1-oxopentan-2-yl]carbamate	Amino acid and its metabolites	C_26_H_41_N_3_O_5_
4-Chlorobenzaldehyde	Benzene and substituted derivatives	C_7_H_5_ClO
Val-Ile-Pro-Lys-Ser	Amino acid and its metabolites	C_25_H_46_N_6_O_7_
Erythrose	Carbohydrates and their metabolites	C_4_H_8_O_4_
Deoxypeganine	Heterocyclic compounds	C_11_H_12_N_2_
(10-Methoxy-1,4,14,19,19-pentamethyl-8,17-dioxo-2,7,18-trioxapentacyclo [11.9.0.03,11.05,9.014,20]docosa-3(11),4,9-trien-21-yl) acetate	Heterocyclic compounds	C_27_H_34_O_8_
2-Cyclohexen-1-one	Aldehyde, Ketones, Esters	C_6_H_8_O

## Data Availability

The raw data used in this study are publicly available on FigShare at DOI: 10.6084/m9.figshare.29923904.
